# 4537 Association between Geographic Socioeconomic Disadvantage and Incidence of Total Hip Replacement Surgery

**DOI:** 10.1017/cts.2020.258

**Published:** 2020-07-29

**Authors:** Rafa Rahman, Joseph K. Canner, Elliot R. Haut, Casey J. Humbyrd

**Affiliations:** 1 Johns Hopkins University School of Medicine

## Abstract

OBJECTIVES/GOALS: Total hip replacement (THR) improves function for those with arthritis, but not all patients have equal access to this elective procedure. To better geographically target healthcare resources, we explored whether geographic socioeconomic disadvantage is associated with incidence of elective THR. METHODS/STUDY POPULATION: We performed a cross-sectional analysis of data in the state of Maryland from 2013-2019. We categorized 5-digit zipcodes into national quartiles of socioeconomic disadvantage using the Area Deprivation Index (ADI). For each zipcode, we calculated the THR incidence rate using Maryland Health Services Cost Review Commission (HSCRC) inpatient and outpatient data in those age 65 years and older. We included only elective THRs. We analyzed the association between a zipcode’s disadvantage quartile and THR incidence rate using multivariate linear regression, correcting for differences across zipcodes in gender, race, and ethnicity distributions, and distance to the nearest hospital performing THRs. RESULTS/ANTICIPATED RESULTS: We analyzed 414 zipcodes with overall average THR rate of 370.8 per 100,000 persons >65yo per year. Relative to zipcodes in the least socioeconomically disadvantaged quartile, those in the second-least disadvantaged had 82.2 fewer THRs per 100,000 persons >65yo per year, those in the second-most disadvantaged had 144.2 fewer, and those in the most disadvantaged had 207.4 fewer (all p65yo per year, those in the second-most disadvantaged had 136.2 fewer, and those in the most disadvantaged had 182.9 fewer (all p <.05). DISCUSSION/SIGNIFICANCE OF IMPACT: More socioeconomically disadvantaged areas have significantly lower rates of elective THR, independent of differences in demographics and hospital proximity. These findings show how disparities can affect access and outcomes, and should inform targeting of community-level education and intervention.

